# An evolving new paradigm: endothelial cells – conditional innate immune cells

**DOI:** 10.1186/1756-8722-6-61

**Published:** 2013-08-22

**Authors:** Jietang Mai, Anthony Virtue, Jerry Shen, Hong Wang, Xiao-Feng Yang

**Affiliations:** 1 Centers of Metabolic Disease Research, Cardiovascular Research, Thrombosis Research, Department of Pharmacology, Temple University School of Medicine, Philadelphia, PA 19140, USA; 2 Department of Family Medicine, College of Community Health Sciences, University of Alabama, Tuscaloosa, AL 35487, USA

**Keywords:** Innate immunity, Endothelial cells, Innate immune cells, Vascular inflammation, Cytokines

## Abstract

Endothelial cells (ECs) are a heterogeneous population that fulfills many physiological processes. ECs also actively participate in both innate and adaptive immune responses. ECs are one of the first cell types to detect foreign pathogens and endogenous metabolite-related danger signals in the bloodstream, in which ECs function as danger signal sensors. Treatment with lipopolysaccharide activates ECs, causing the production of pro-inflammatory cytokines and chemokines, which amplify the immune response by recruiting immune cells. Thus, ECs function as immune/inflammation effectors and immune cell mobilizers. ECs also induce cytokine production by immune cells, in which ECs function as immune regulators either by activating or suppressing immune cell function. In addition, under certain conditions, ECs can serve as antigen presenting cells (antigen presenters) by expressing both MHC I and II molecules and presenting endothelial antigens to T cells. These facts along with the new concept of endothelial plasticity suggest that ECs are dynamic cells that respond to extracellular environmental changes and play a meaningful role in immune system function. Based on these novel EC functions, we propose a new paradigm that ECs are conditional innate immune cells. This paradigm provides a novel insight into the functions of ECs in inflammatory/immune pathologies.

## Introduction

Endothelial cells (ECs) form a single cell layer called the endothelium, which lines the vasculature and lymphatic systems forming a semi-permeable barrier between blood or lymph within vessels and the surrounding tissues. The endothelium is a highly specialized, dynamic, disseminated organ with many essential functions in physiological processes. In its entirety, the endothelium is composed of 1 to 6 × 10^13^ ECs covering a surface area of more than 1000 square meter [1]. ECs from different vascular sites also have numerous variations in their appearance. Vascular ECs usually have a flattened squamous structure, but they can also be cuboidal and have varying thicknesses from less than 0.1 μm to 1 μm across the vascular tree. Moreover, ECs among different tissues are heterogeneous with respect to their protein and surface marker expressions [2,3]. In fact, different subsets of ECs can be found within a single organ such as the liver [4]. The heterogeneity of ECs contributes to their diversity in function at different vascular sites [5-7].

Besides serving as a physical barrier, ECs have a wide array of functions which are characterized into three major categories: trophic, tonic, and trafficking [8]. Under physiological conditions, ECs are involved in the modulations of metabolic homeostasis (trophic function), vascular hemodynamics (tonic function), vascular permeability, coagulation, and cell extravasation (trafficking) [8]. In a quiescent state, ECs balance the release of various vasodilating or vasoconstricting factors such as nitric oxide, prostacyclins, and endothelin to maintain vascular tone, blood pressure, and blood flow [9]. Furthermore, the endothelium is crucial in regulating coagulation, utilizing both anti-coagulation and pro-coagulation mechanisms. Under standard physiological conditions ECs express inhibitors of the tissue factor pathway and thrombomodulin, which prevents the activation of pro-coagulation molecules including factor X, thrombin, and fibrin. However, once the endothelium is injured, the EC surface quickly transforms to a pro-coagulant state by inducing tissue factors that initiate the extrinsic coagulation cascade [10]. In addition to coagulation, ECs have an essential role in modulating vascular permeability. This function regulates the ability of cells to move to and from the circulatory system during inflammatory responses. Under normal physiological conditions, endothelium basal permeability only allows for the easy diffusion of solutes such as glucose, ions, and other metabolites to underlying cells. However, during states of acute and chronic inflammation, endothelial permeability is increased, allowing for additional trafficking of immune cells. Excessive or prolonged increases in permeability, as seen in cases such as chronic inflammation, can have deleterious effects resulting in tissue edema. Endothelial permeability is mediated via two pathways; paracellular and transcellullar [11,12]. The inability of ECs to adequately carry out these or any other basal functions is referred to as endothelial dysfunction, and is a hallmark of several cardiovascular diseases.

In addition to the aforementioned physiological functions, ECs also have important immunological functions. Cells of the immune system function to defend against invasive foreign pathogens and detrimental endogenous materials. Cells of the innate immune system cells include neutrophils, monocytes, macrophages, dendritic cells (DCs), Langerhans cell, natural killer (NK) cells, basophils, mast cells, and eosinophils, whereas cells of the adaptive immune system are comprised of B, T and NKT lymphocytes. The innate immune system mediates non-specific immunity, thus its response is immediate and antigen-independent. Innate immune cells patrol the blood and are the first to sense foreign pathogens, acting as a barrier to infections. Upon pathogen detection, cells of the innate immune system produce cytokines and chemokines which recruit phagocytes to the site of infection. Phagocytes, including neutrophils and macrophages, engulf and destroy foreign pathogens via granules or lysosomes that contain proteolytic and hydrolytic enzymes. On the other hand, the initiation of adaptive immunity requires the interaction of innate immune cells with cells of the adaptive immune system. Professional antigen-presenting cells, such as macrophages, DCs, and B cells, engulf pathogens and then process and present peptide antigens to lymphocytes via major histocompatibility complex (MHC) class I and class II molecules. Although B cells can recognize and respond to membrane-bound and non-membrane-associated antigens, it has been suggested that membrane-associated antigens are more essential for B cell activation than soluble antigens *in vivo*[13]. Innate immune cells that patrol the blood, such as DCs, are equipped with a series of pathogen-associated molecular pattern receptors including Toll-like receptors (TLRs) [14] and nucleotide-binding oligomerization domain (NOD)-like receptors (NLRs) [15]. TLRs, NLRs, retinoic acid inducible gene 1 (RIG-I)-like receptors (RLRs), absent in melanoma 2 (AIM2)—like receptors (ALRs) and C-type lectin receptors (CLRs) are pattern recognition receptors (PRRs), which can identify pathogen-associated molecular patterns (PAMPs). These receptors are part of the innate immune system and are known to be expressed on immune cells as well as non-immune cells [16]. PRRs are able to sense components of exogenous microbes as well as harmful endogenous components. In addition to detecting different pathogens and secreting cytokines [17], DCs are also equipped with cytokine and chemokine receptors, which allow DCs to differentiate and mature in response to their environment [18].

### Endothelial cells are sentinels of the innate immune system

Due to their location, ECs are one of the first cells to interact with microbial components in the circulation. Therefore, it can be extrapolated that EC recognition and response may be integral to early innate immune system activation. In fact, like DCs, ECs are reported to express both TLRs and NLRs [19,20], as well as express chemokine receptors [21,22]. Specifically, ECs have been shown to secrete the pro-inflammatory cytokine interleukin-8 (IL-8) in a NOD1-dependent manner in response to microbial stimulation [23,24]. In addition, ECs have been reported to express the NOD2 receptor, which recognizes the bacterial peptidoglycan muramyl dipeptide [25]. Muramyl dipeptide can activate ECs, leading to the upregulation of IL-6 secretion, which induces CD4+ T helper cell-17 (Th17) polarization while inhibiting CD4+ Th1 and Th2 responses [26].

Immune responsive ECs in healthy arteries express low levels of TLR2 and TLR4, whereas inflamed endothelium and endothelium of atherosclerotic lesions have significant upregulation of TLR2 and TLR4 expression [27]. Lipopolysaccharide (LPS) is a major component of Gram-negative bacteria cell wall that has been shown to induce EC responses such as the production of IL-1, IL-8, and monocyte chemotactic protein-1 (MCP-1) via TLR4 [28-31]. Similarly, LPS, tumor necrosis factor-α (TNF-α), and interferon-γ (IFN-γ) can induce TLR2 expression via a NF-κB-dependent manner, highlighting the importance of TLR2 in innate immunity and host defense against Gram-positive cell wall components [32]. It should be noted that ECs also express CD14 which is also a known receptor for LPS [33]. TLR3, TLR7, and TLR8 are important in detecting viral RNA and activating innate immune responses against viruses. Although TLR7 and TLR8 are not detected in ECs, human umbilical vein endothelial cells (HUVECs) do express TLR3. In fact, ECs also express IFN-α, which is an important cytokine in regulating innate immune responses against viruses and is shown to strongly induce TLR3 expression [34]. Moreover, ECs also express TLR9 which recognizes viral and bacterial DNA [27,35].

Aside from the expression of PRRs, ECs also express important downstream adaptor molecules for PRR signaling including myeloid differentiation-2 (MD2) and myeloid differentiation primary-response protein 88 (MyD88). MD2, also known as lymphocyte antigen 96 (Ly96), is a protein associated with the extracellular domain of TLR-4 that is required for LPS signaling through TLR-4 [36,37]. Meanwhile, MyD88 is an important intracellular adaptor molecule in the canonical TLR signaling cascade.

Under physiological conditions, ECs express the lectin-like oxidized low density lipoprotein (oxLDL) receptor (LOX-1) at low levels. Strong evidence has suggested a pathological role of LOX-1 in atherosclerosis, a chronic autoimmune inflammatory disease. ECs have been shown to upregulate the expression of LOX-1 in response to stimulation by oxLDL, pro-inflammatory cytokines, and proatherogenic factors such as angiotensin II [38].

In addition to enhanced expression of LOX-1 by ECs, oxLDL also induces endothelial activation-featured cell surface adhesion molecule expression [39,40] and has been shown to impair nitric oxide (NO) production in ECs by increasing superoxide generation [41]. An additional function of LOX-1 is the mediation of endothelial phagocytosis of aged red blood cells and apoptotic cells that express phosphatidylserine on the cell surface. It should also be noted that this LOX-1-mediated phagocytotic activity can be inhibited by oxLDL. In addition, the expression of phosphatidyleserine on the cell surface is reported to have pro-coagulation activity. Thus, LOX-1 is important in endothelial-mediated vascular homeostasis and coagulation prevention under physiological conditions [42].

### Endothelial cells are conditional antigen presenting cells

The adaptive immune response is triggered when innate immunity fails to eliminate inflammatory stimuli resulting in the progression of the inflammatory reaction from one that is acute to one that is chronic. The endothelium participates in chronic inflammation via interactions with specialized effector cells and by acting as antigen presenting cells (APCs) [43]. Although ECs are not professional APCs, their secondary role in antigen presentation has been recognized [44,45].

The participation of ECs in antigen presentation was first indicated with the discovery of both MHC class I and class II molecule expression. MHC I is expressed by all nucleated cells, with basal levels being detected in ECs [46]. Unlike the expression of MHC I by all cell types, MHC II expression is limited to APCs [47]. Professional APCs ubiquitously express MHC II, while cells such as ECs, which are not considered classic APC, can induce MHC II expression [48]. Moreover, MHC II molecules are also found to be basally expressed in the microvasculature [49]. In response to stimulations such as hydrogen peroxide and IFN-γ, ECs can upregulate the expression of MHC I and induce the expression of MHC II [46,48,50]. In addition, activated ECs also express co-stimulators including 4-1BB ligand (4-1BBL), inducible co-stimulator ligand (ICOSL), and OX40 ligand (OX40L), which are involved in memory T cell formation, activation, and survival [51,52]. Meanwhile, ECs treated with IFN-γ effectively induce CD4+ and CD8+ memory T cells to produce cytokines and proliferate [52].

In mice, sinusoidal ECs expressing MHC molecules and co-stimulators B7-1 and B7-2 are found to have effective antigen presenting function *in vitro*[53]. B7-1 and B7-2, also known as CD80 and CD86, respectively, are used by professional APC to provide co-stimulation to T cells via interaction with T cell CD28. Furthermore, the glomerular endothelium also expresses B7-1 and B7-2 co-stimulators in ischemia/reperfusion injury, an event that inevitability occurs in organ transplantation. The notion that ECs act as APCs in organ transplant is highlighted in studies where endothelium MHC and co-stimulator molecule expressions are shown to trigger allogeneic and autoimmune responses by memory T cells leading to allograft rejection [52,54,55].

ECs have also been shown to express and up-regulate CD1d upon stimulation [56,57]. CD1d is an MHC class I-like molecule expressed by APCs and non-hematopoietic cells. Cells expressing CD1d present glycolipid antigen to invariant natural killer T cells (iNKT) during infection resulting in their activation [58]. Although ECs express CD1d and may have the capacity to present antigen to iNKT, evidence of this has yet to be obtained *in vivo*.

The aforementioned expressions of MHC molecules and co-stimulators by ECs selectively regulate the migration of antigen-specific lymphocytes to sites of inflammation [44]. One of the first studies to show that antigen presentation by ECs influences T cell recruitment was seen in the guinea pig experimental allergic encephalomyelitis model. In this model, central nervous system ECs were shown to increase Ia antigen presentation before inflammatory cell infiltration. Furthermore, clinical signs were detected that suggest endothelial Ia presentation is involved in T cell recruitment in the disease [59]. Other *in vitro* data showed that transendothelial migration of antigen-specific T cells is enhanced across ECs that express that specific antigen. The frequency of T cells with antigen specificity for MHC class II-DR17 transmigrate across an endothelial monolayer that expresses DR17 antigen at a fourfold higher rate than other migrating T cells [60]. In type I diabetes, ECs are shown to have a capacity to process and present islet autoantigen glutamic acid decarboxylase GAD65 to autoreactive T cells and enhance the transmigration of GAD65-specific T-cells [61]. Moreover, pancreatic ECs are able to present insulin with MHC class I to activated insulin-specific CD8+ T cells. This causes their infiltration into the pancreas, leading to beta cell destruction and the onset of diabetes [62]. Endothelium antigen recognition by T lymphocytes is also shown to drive the recruitment and tissue infiltration of T cells *in vivo*. In fact, T cell and EC interactions were visualized *in vivo* by intravital microscopy. In a study it was shown HY antigen (a male tissue specific antigen) presentation by the endothelium enhanced HY-specific CD8+ T cells transendothelial cell migration resulting in a large influx of T cells into tissues [63]. It is also reported that the trafficking of antigen-specific CD8+ T cell across the blood brain barrier into the brain depends on cerebral endothelium expression of MHC I. It was shown that antigen-specific CD8+ T cells only infiltrated into the brain when their cognate antigen was present. Moreover, when antibody against MHC I was used, CD8+ T cell infiltration was significantly reduced [64].

Antigen presentation is known to be one of the first steps in initiating adaptive immunity; however, in particular circumstances antigen presentation can also induce immune tolerance. Under physiological conditions, MHC I antigen presentation by liver sinusoidal endothelial cells (LSECs) leads to recruitment of antigen-specific naïve CD8+ T cells and the induction of local tolerance [65]. In addition, LSECs are shown to cross-present antigen to CD8+ T cells at a relatively low concentration compared to myeloid APCs, such as macrophages and DCs. In fact, CD8+ T cells co-cultured with antigen-presenting LSECs secrete IFNγ and IL-2; however, upon re-stimulation, the ability to secrete IFNγ and IL-2 is diminished. Furthermore, CD8+ T cells had impaired cytokine expression with extended co-culture [66]. Antigen-presenting LSECs also have the ability to prime naïve CD4+ T cells but fail to induce T effector cell differentiation as seen with priming by other APCs [67]. Instead, LSEC-primed naïve CD4+ T cells acquired regulatory properties marked by suppression of naïve CD4+ responder T cell proliferation *in vitro* and suppression of inflammation in an ovalbumin (OVA)-specific autoimmune hepatitis model [68].

### Immune enhancing and immune suppressive roles of endothelial cells

**Figure 1:**
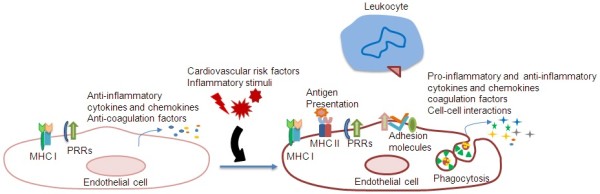
**Endothelial cells are conditional innate immune cells.** In their quiescent state, endothelial cells express MHC I (Major histocompatibility class I) molecules and PPRs (pattern-recognition receptors) which detect PAMPs (pathogen-associated molecular patterns). In the presence of inflammatory stimuli and risk factors in the bloodstream, endothelial cells transform from an anti-inflammatory and anti-coagulatory state to a pro-inflammatory and pro-coagulatory state. Endothelial cells can detect inflammatory stimuli and risk factors via PRRs. In response to these stimuli, endothelial cells express MHC II molecules which present endothelial antigens to immune cells. Moreover, endothelial cells can upregulate the expression of surface adhesion molecules that induce the adhesion of immune cells, such as leukocytes, to the endothelium and facilitate transmigration to underlying tissues. In addition, endothelial cells enhance the secretion of pro-inflammatory cytokines and chemokines which can modulate the activities of immune cells.

ECs can either have immune enhancing or suppressive functions depending on their cytokine profile and their interaction with other immune cells. Cytokines are small signaling molecules, secreted by cells, which can modulate the behavior and properties of cells via autocrine, paracrine, or endocrine mechanisms. Cytokines also function to regulate immune responses. The location of ECs makes them one of the first targets of cytokines circulating in the blood stream. It should be noted, however, ECs are not merely targets of cytokines, they also have the capacity to generate and secrete cytokines under certain circumstances (Figure 1, Table 1).

**Table 1:** List of pro-inflammatory/anti-inflammatory cytokines and chemokines as well as growth factors produced by endothelial cells in response to various stimuli

**Cytokines**	**Stimuli**	**Endothelial cells**	**References**
IL-1α	TNF-α	HCAEC	[69]
	TNF-α, IL-1β, LPS, TCC, LPS, hypoxia	HUVEC	[31,69-72]
	*Trypanosoma cruzi* infection	HUVEC	[73]
	TNF-α	HPAEC	[69]
	TNF-α, TCC	HIMEC	[70]
	Basal	MBEC	[74]
IL-1β	TCC, LPS, hypoxia,	HUVEC	[31,70,72]
	*Trypanosoma cruzi* infection	HUVEC	[73]
	IL-1β, TCC	HIMEC	[70]
	Shear stress	BAEC	[75]
	LPS	HCAEC	[76]
IL-3	Basal	HUVEC, HIMEC	[70]
IL-5	Basal	HCAEC, HUVEC, HPAEC	[69]
IL-6	TNF-α, LPS	HCAEC	[69,76]
	TNF-α, IL-1β, IL-4, IFN-γ, TCC, hypoxia	HUVEC	[69,70,72]
	*Trypanosoma cruzi* infection	HUVEC	[73]
	IL-4 + IL-1β	HUVEC	[77]
	Histamine	HUVEC	[78]
	LPS	HUVEC	[79]
	IL-4 or IFN-γ + TNF-α or IL-1β	HUAEC	[80]
	TNF-α + IL-4, TNF-α + IFN-γ	HSVEC	[80]
	TNF-α	HPAEC	[69]
	TNF-α, IL-1β, IL-4, IFN-γ, TCC	HIMEC	[70]
	Shear stress	BAEC	[75]
	Basal, LPS	MBEC	[74]
IL-8	TNF-α, LPS	HCAEC	[69,76]
	TNF-α, IL-1β, IL-4, IFN-γ, TCC	HUVEC	[69,70,81]
	TNF-α	HPAEC	[69]
	TNF-α, IL-1β,TCC	HIMEC	[70]
	Oral viridian streptococci	HSVEC	[82]
IL-10	Basal	MBEC	[74]
IL-11	Basal, PMA	HUVEC	[70,83]
	Basal	HIMEC	[70]
	Basal, PMA	HAEC	[83]
G-CSF	MM-LDL	HAEC, RAEC	[84]
GM-CSF	TNF-α	HCAEC, HPAEC	[69]
	TNF-α, IL-1β, TNF-α + IL-4	HUVEC	[69,70,80]
	TNF-α + IL-4	HUAEC, HSVEC	[80]
	TNF-α, IL-1β	HIMEC	[70]
	MM-LDL	HAEC, RAEC	[84]
	Basal, LPS	MBEC	[74]
MCP-1	IL-4+ IL-1β, IL-4 + LPS	HUVEC	[77]
(CCL2)	LPC	HUVEC	[85]
	LPS	HCAEC	[76]
M-CSF	MM-LDL	HAEC, RAEC	[84]
	*Trypanosoma cruzi* infection	HUVEC	[73]
RANTES	TNF-α + IFN-γ	HUVEC	[86]
(CCL5)	TNF-α + IFN-γ, IL-1β	HMMEC	[87]
TGF-β	TNF-α, IL-1β	HUVEC, HIMEC	[70]
TNF-α	TNF-α, IL-1β	HUVEC, HIMEC	[70]
	Basal	MBEC	[74]
	LPS	HCAEC	[76]

Cytokine/chemokine induction either on mRNA or protein level.

Basal: basal expression without stimulus; Human coronary artery endothelial cell: HCAEC; Human umbilical vein endothelial cell: HUVEC; Human pulmonary artery endothelial cell: HPAEC; human intestinal microvascular endothelial cell: HIMEC; Gulten reactive T cell clones supernatant: TCC; phorbol 12-myristate 13-acetate: PMA; Human saphenous vein endothelial cell: HSVEC; Human aortic endothelial cell: HAEC; Rabbit aortic endothelial cell: RAEC; Minimally modified low density lipoprotein: MM-LDL; Bovine aortic endothelial cell: BAEC; Lysophosphatidylcholine: LPC; Human mucosal microvascular endothelial cell:HMMEC; Mouse brain endothelial cell: MBEC.

Various factors and stimuli, including cytokines, can induce EC activation, a state of heightened responsiveness. EC activation may be classified into two types, type I and type II. Type I activation includes rapid responses that are independent of new gene expression. These responses are mediated by ligands binding to the extracellular domains of heterotrimeric G protein-coupled receptor (GPCRs) and signal through the intracellular G-protein αq subunit. Type II activation is a relatively slower response that depends on new gene expression but delivers a more sustained inflammatory response [88]. EC activation is integral in mediating a proper inflammatory response; however, prolonged activation can also lead to endothelial inflammation and dysfunction which precede the development of several vascular diseases. Once activated, ECs can upregulate cell surface adhesion molecules and pro-thrombotic molecules. Moreover, activated ECs can generate and secrete pro-inflammatory cytokines and chemokines. In their basal state, cultured ECs have detectable mRNA levels of numerous pro-inflammatory and anti-inflammatory cytokines including IL-3, IL-7, IL-8, IL-11, IL-15, TNF-α, and transforming growth factor-β (TGF-β) [70]. However, the expression of pro-inflammatory cytokines is marginal, possibly inhibited by basal production of NO which maintains endothelium quiescence [89]. Meanwhile, in response to pro-inflammatory stimuli such as hypoxia, infection, or oxLDL, ECs upregulate the production of cytokines and chemokines [90].

#### Pro-inflammatory

The broad spectrum of pro-inflammatory cytokines and chemokines expressed by ECs includes IL-1β, IL-3, IL-5, IL-6, IL-8, IL-11, IL-15, growth-regulated oncogene α (GRO-α, CXCL1), MCP-1(CCL2), RANTES(CCL5) , and TNF-α [90,91]. These pro-inflammatory cytokines and chemokines are important in potentiating inflammatory responses by inducing cytokine secretion by other cells and recruiting immune cells to the site of inflammation. Cytokines IL-1 and TNF-α are particularly effective in inciting the expression of pro-inflammatory genes in various cells. In addition, IL-1 and TNF-α synergistically promote the inflammatory process, especially when induced by infections, trauma, ischemia, immune-activated T cells, or toxins [92]. Moreover, in response to stimuli, the endothelium is also capable of expressing various growth factors including granulocyte colony-stimulating factor (G-CSF), macrophage colony-stimulating factor (M-CSF), granulocyte-macrophage colony-stimulating factor (GM-CSF), platelet-derived growth factor (PDGF), vascular endothelial growth factor (VEGF), and fibroblast growth factors (FGF) [93]. These factors play a critical role during wound healing, menstrual cycles, cancers, and various ischemic and inflammatory diseases [94]. In addition, colony-stimulating factors and growth factors produced by the endothelium are important for hematopoiesis which increases the number of immune cells in the circulation during inflammation.

Aside from promoting inflammatory responses via cytokine production, ECs also physically interact with immune cells during the inflammatory process. ECs at rest do not interact with leukocytes; however, during inflammation, activated ECs upregulate the expression of adhesion molecules and chemokines [95]. Adhesion molecules including P-selectin, E-selectin, vascular cell adhesion molecule-1 (VCAM-1), and intercellular adhesion molecule-1 (ICAM-1) are upregulated leading to leukocyte transmigration across the endothelium to the site of inflammation. Leukocytes are recruited to the endothelium at the site of injury via a series of steps called the leukocyte adhesion cascade. Adhesion molecules and chemokines are important in mediating each step of this cascade. Initially, chemokines from the endothelium attract and activate leukocytes. Then, leukocytes will tether to and roll on EC surface during the initial step of extravasation during inflammation. Leukocytes will then undergo activation leading to arrest and spreading on the endothelium. While the tethering and rolling on ECs are mediated by selectins, the arrest and spreading of leukocytes are mediated by integrins [96]. Finally, mediated by platelet endothelial cell adhesion molecule-1 (PECAM-1, CD31) and ICAM-1, leukocytes will transmigrate between ECs to the underlying tissues.

Like the expression of MHC and co-stimulator molecules which selectively regulate the influx of antigen-specific cells to the site of injury, adhesion molecules and chemokines also facilitate this function. The endothelium expression of particular adhesion molecules and chemokines changes when acute inflammation progresses into chronic inflammation, thus allowing for the extravasation of different effector cells. The selective influx of effector cells permits for the polarization of immune responses in adaptive immunity. During inflammation that is dominated by Th1 cells, ECs preferentially express chemokine (C-X-C motif) ligand 10 (CXCL10) and E-selectin which favor the recruitment of Th1 cells [97]. Meanwhile, ECs express chemokine (C-C motif) ligand 26 (CCL26) and VCAM1 to favor recruitment of Th2 cells during an immune response that is dominated by Th2 cells [98]. Furthermore, ECs can secrete the chemokine GROα or MCP-1 to attract neutrophils or monocytes respectively, during an acute inflammatory process [99,100].

Besides regulating immune cell recruitment to specific inflammation sites, ECs can also signal immune cells to produce cytokines. This induction occurs not only through endothelial cytokine signaling but also by direct physical interactions. EC surface molecules such as lymphocyte function-associated antigen (LFA)-3 and ICAM-1 have been shown to signal and increase IL-2 and IL-4 production by T cells. In addition, co-culture of ECs with activated T cells enhanced IFN-γ production. It has also been shown that T cells are more responsive to IL-12 stimulation in the presence of EC co-culture [101]. Mechanistically, ECs have been found to increase and prolong cytokine production by T cells via OX40 signaling. Co-stimulation of OX40 is shown to stabilize the mRNAs of IL-2, IL-3, and IFN-γ [102]. These data suggest that ECs have co-stimulatory signals that are necessary for optimal T cell activation. EC co-stimulation effectively triggers cytokine secretion from naïve and memory CD4+ T cells; however, it should be noted that co-stimulation does not seem to induce differentiation of human naïve CD4+ T cells [103].

Preliminary work has also demonstrated the impact of ECs on DC maturation. An anti-angiogenic cytokine derived from ECs, vascular endothelial growth inhibitor functions to suppress EC proliferation in a cell cycle-dependent manner. Aside from its impact on ECs, vascular endothelial growth inhibitor has also been revealed to promote the maturation of mouse DCs, which is an important step in the initiation of adaptive immunity [104].

#### Anti-inflammatory

Besides pro-inflammatory cytokines, ECs can also produce anti-inflammatory cytokines such as IL-1 receptor antagonist (IL-1ra), IL-10, IL-13, and TGF-β [91]. Mechanistically, anti-inflammatory cytokines can either block the process initiated by pro-inflammatory cytokines or suppress the progression of the inflammatory cascade. For example, cytokines such as IL-4, IL-10, IL-13, and TGF-β suppress the production of IL-1, TNF-α, and other pro-inflammatory cytokines [92]. In fact, the balance between pro-inflammatory and anti-inflammatory cytokines is believed to decide the result of inflammatory disease progression. It has been postulated that the propensity for developing inflammatory diseases is determined by the dominant expression of pro-inflammatory cytokines or the inadequate expression of anti-inflammatory cytokines. Examples include IL-10-deficient mice which develop inflammatory bowel disease, TGF-β1 knockout mice getting spontaneous inflammatory disease, and mice deficient in IL-1ra obtaining a disease that is nearly identical to rheumatoid arthritis [92].

Aside from aiding T cells in playing a pro-inflammatory role in immune responses, ECs can also induce suppressive immune function in T cells. Mouse ECs activated by IFN-γ and co-cultured with allogeneic CD4+ T cells were shown to induce the generation of CD4+ CD25+ FOXP3+ regulatory T cells. Further analysis of this regulatory T cell population revealed the upregulation of surface glucocorticoid-induced TNFR-related protein (GITR) and intracellular cytotoxic T-Lymphocyte Antigen 4 (CTLA4), suggesting that these CD4+ CD25+ FOXP3+ regulatory T cells were activated and could inhibit the proliferation of alloreactive T cells [105]. Furthermore, it was found that co-culture of regulatory T cells with ECs can enhance the suppressive function of CD4+ CD25+ FOXP3+ regulatory T cells. Mechanistically, after contact with ECs, regulatory T cells upregulate the expression of programmed death-1 receptor and increase the production of anti-inflammatory cytokines IL-10 and TGF-β [106].

### Endothelial-derived microparticles

In addition to cellular mediators such as cytokine, chemokines, and adhesion molecules, endothelial cells also induce cellular signaling via microparticles. Microparticles are small plasma membrane-derived vesicles, usually 0.1-1.5 μm in diameter, that are released by various cell types during cell activation or apoptosis - a type of programmed cell death [107]. Various factors are shown to induce endothelial vesiculation (microparticle formation) *in vitro*, including TNF-α, IL-1β, thrombin, calcium ionophore [108], and reactive oxygen species [109]. Endothelial microparticles (EMPs) are also released in the absence of stimulation, and microparticles of various cellular origins are detected in healthy individuals, suggesting that microparticle formation is a physiological process. EMPs are found to be increased in patients with various cardiovascular related diseases, including acute coronary syndrome, hypertension, and heart failure [110]. Furthermore, in patients with systemic lupus erythematosus, a risk factor of cardiovascular disease, higher levels of EMPs were found. In fact, after receiving immunosuppressive treatments to control their inflammatory disease activity, these patients were shown to have reduced EMPs levels [111]. It should be noted that circulating EMPs are not merely biomarkers of inflammatory diseases but also contributes the pathological state.

Microparticles express surface antigens from their cells of origin which allows for the identification of their sources. Depending on the stimulus which triggers their release, EMPs may contain endothelial proteins such as ICAM-1, PECAM-1, ανβ3 integrin, and VE-Cadherin [112]. Moreover, EMPs also have endothelial nuclear materials such as microRNA, RNA, and DNA. It has been shown that EMPs can induce intracellular signaling via the transfer of these nuclear materials and proteins to target cells [113]. EMPs are also found to have pro-coagulant and pro-adhesive properties [108], which promote coagulation and vascular inflammation. In fact, increased levels of circulating EMPs were detected in patients with diabetes. It was found that EMPs generated by high glucose treated cells, but not control untreated cells, induced vascular inflammation and endothelial dysfunction via activation of p38 by NADPH oxidase [114]. EMPs also induced inflammation in acute lung injury by increasing pulmonary and systemic levels of IL-1β and TNF-α. These elevated pro-inflammatory cytokine levels were found to correlate with increased neutrophil recruitment to the lungs [115]. EMPs were also found to induce the maturation of plasmacytoid dendritic cells whereas microparticles from T cells or platelet did not, under the same conditions studied. Plasmacytoid dendritic cells matured by EMPs were shown to secrete pro-inflammatory cytokines, IL-6 and IL-8, and induced proliferation of allogeneic naïve CD4+ T cells [116].

### Endothelial cell plasticity

Naïve T cells can differentiate into different mature T cells depending on the integration of a number of factors. Previously, it has been proposed that activated T cells are committed to a specific terminal T cell phenotype. However, as our knowledge in the field advances, we have come to realize that activated T cells are flexible in their cytokine production in response to various stimuli. The plasticity of T cell subsets allows for rapid immune system response to various pathogens [117]. Similar to classic immune cells, ECs were initially thought to be a terminally differentiated cell type. Recently, however, the concept of EC plasticity has gained recognition. During embryogenesis, ECs derived from the mesoderm, via vasculogenesis, give rise to the early embryonic vasculature. Then through angiogenesis, this primitive vasculature network remodels and forms new blood vessels. As the vasculature develops, ECs become differentiated into either arterial or venous specific cells. Notch signaling is crucial during this process, while several transcription factors have been identified that also play a role in this development [118]. Specifically, Notch signaling is important in promoting EC differentiation toward arterial cell differentiation suppressing venous cell development. This is further supported by the fact that chicken ovalbumin upstream promoter-transcription factor II (COUP-TFII), an orphan nuclear receptor, suppresses Notch signaling and thereby promotes venous cell differentiation [119].

It has been well cited that blood ECs can be reprogramed to lymphatic EC and vice versa via the activity of homeobox transcription factor Prox1 [120-122]. The lymphatic vasculature works in concert with the blood vasculature in maintaining fluid homeostasis by collecting fluid from tissues and returning it to the blood supply. More importantly, the lymphatic vasculature transports immune cells such as APCs to the lymph nodes where immune responses can be initiated. The mammalian lymphatic vasculature is venous-derived as indicated by lineage-tracing [123], suggesting that the existence of a normal blood vascular network is a criterion for the lymphatic vasculature. It has been proposed that the plasticity of ECs via Prox1 regulation allows for the rapid formation of blood ECs by dedifferentiation of lymphatic ECs. Under conditions of rapid demand for additional blood supply, the formation of new vessels from bone marrow-derived endothelial progenitor cells may not be quick enough. In order to satisfy this demand, the dedifferentiation of nearby lymphatic EC could speedily provide additional blood ECs [122]. Reciprocally, during inflammatory processes where the rapid utilization of immune cells requires the lymphatic system, blood ECs that are infected with Kaposi’s sarcoma-associated herpesvirus (a chronic inflammatory condition) has been shown to switch into a lymphatic EC phenotype [124]. Exposure of inflammatory cytokines to blood ECs has been shown to have a similar effect [125].

In addition to differentiation into alternative EC lineage, ECs may also be induced to have stem-cell like properties via endothelial-mesenchymal transition (EndMT). During EndMT, mature and progenitor ECs acquire a mesenchymal phenotype that can give rise to other cell types [126,127]. EndMT is crucial during embryonic development. When the heart develops, some ECs that line the endocardial cushion go through EndMT. Some of these ECs remain vascular, while others become mesenchyme and enter the underlying tissue to participate in the formation of the heart valves and septa [128]. Mechanistically, TGF-β and Notch pathways have been shown to be important in regulating endothelial plasticity [129]. The role of EndMT in disease progression has been postulated and specifically in cancer has been explored. It was found that EndMT is a contributing source of cancer-associated fibroblasts, cells that participate in tumor growth and metastasis [130]. In addition to cancer, ECs are also found to be a source of fibroblasts through EndMT. These cells have a pathological impact by contributing to the fibrosis of organs such as the kidney, lung, and heart [131-133].

### Summary

ECs are a heterogeneous population that carries out many essential physiological processes. Aside from these basal functions, ECs also actively participate in both innate and adaptive immunity. Due to their location, ECs are one of the first cell types to detect foreign pathogens and endogenous metabolite-related danger signals in the bloodstream. Treatment with bacterial endotoxin such as LPS activates ECs causing the production of pro-inflammatory cytokines and chemokines, which amplify the immune response by attracting and mediating the extravasations of immune cells. Besides immune cell trafficking, ECs also induce cytokine production in immune cells. In addition, under certain conditions, ECs can serve as antigen presenting cells (Table 2). In fact, expressing both MHC I and II molecules, it has been shown that recognition of endothelial antigens by T cells expedites their infiltration in tissues. These facts along with the new concept of endothelial plasticity suggest that ECs are dynamic cells that respond to extracellular environmental changes and play a meaningful role in immune system function.

**Table 2:** Comparison of endothelial cells and macrophages, professional immune cells

	**Endothelial cells**	**Macrophages**
Cytokine secretion	Pro-inflammatory cytokines	Pro-inflammatory cytokines
	Anti-inflammatory cytokines	Anti-inflammatory cytokines [134]
Phagocytic function	Non-professional phagocytic cells	Professional phagocytic cells [135]
	Phagocytosis of age blood cells and apoptotic cells [42]	
Antigen presentation	Non-professional antigen presenting cells [45]	Professional antigen presenting cells [136]
PAMPs and DAMPs sensing	Lectin-like oxidized low-density lipoprotein receptor 1 (LOX-1) [137]	TLRs [138], NLRs [139]
		C-type lectin receptors (CLRs) [140]
	Toll-like receptors(TLRs) [20]	Scavenger receptor Class A Type I and II (SR-A I/II) [141]
	NOD-like receptors(NLRs) [20]	Mannose receptors [143]
	CD36 [142]	Dendritic cell-specific ICAM3-grabbing non-integrin (DC-SIGN) [144]
		Macrophage receptor with collagenous structure (MARCO) [145]
		Complement receptor 3 (CR3) [146]
		CD1 [147], CD14 [148], CD36 [149]
Pro-inflammatory	Produce pro-inflammatory cytokines	Classically activated macrophages [134]
Immune-enhancing	Express adhesion molecules and chemokines to attract circulating leukocytes	Produce high levels of pro-inflammatory mediators and cytokines
Anti-inflammatory	Express inhibitors of the tissue factor pathway and thrombomodulin, which prevents the activation of pro-coagulation pathway	Regulatory macrophages [134]
Immunosuppression		Produce anti-inflammatory cytokine IL-10; limit inflammation during later stages of immune responses
	Augment suppressive function of regulatory T cells	
Migration	Essential for vascular development and angiogenesis [150]	Migration to sites of infection or injury in response to pro-inflammatory stimuli and insults [151,152]
Heterogeneity	Within and among tissues, they may have difference in appearance and variation protein and surface marker expressions	Anatomical locations and functions determine subpopulations
		Surface marker expression overlaps between different subsets [134]
Plasticity	Phenotypic change is dependent on environment and pathological conditions	Phenotypic change is dependent on environment and pathological conditions [134]

## Abbreviations

ECs: Endothelial cells; DCs: Dendritic cells; NK: Natural killer; MHC: Major histocompatibility complex; TLRs: Toll-like receptors; NLRs: Nucleotide-binding oligomerization domain (NOD)-like receptors; RLRs: retinoic acid inducible gene 1 (RIG-I)-like receptors; ALRs: Absent in melanoma 2 (AIM2)—like receptors; CLRs: C-type lectin receptors; PRRs: Pattern recognition receptors; PAMPs: Pathogen-associated molecular patterns; IL: Interleukin; Th: T helper cells; LPS: Lipopolysaccharide; MCP-1: Monocyte chemotactic protein-1; TNF: Tumor necrosis factor; IFN: Interferon; HUVECs: Human umbilical vein endothelial cells; MD2: Myeloid differentiation-2; MyD88: Myeloid differentiation primary-response protein 88; oxLDL: Oxidized low density lipoprotein; LOX-1: Lectin-like oxLDL receptor; NO: Nitric oxide; APCs: Antigen presenting cells; 4-1BBL: 4-1BB ligand; ICOSL: Inducible co-stimulator ligand; OX40L: OX40 ligand; iNKT: Invariant natural killer T cells; LSECs: Liver sinusoidal endothelial cells; GPCR: G protein-coupled receptor; TGF: Transforming growth factor; GRO: Growth-regulated oncogene; CXCL: Chemokine (C-X-C motif) ligand; CCL: Chemokine (C-C motif) ligand; G-CSF: Granulocyte colony-stimulating factor; M-CSF: Macrophage colony-stimulating factor; GM-CSF: Granulocyte-macrophage colony-stimulating factor; PDGF: Platelet-derived growth factor; VEGF: Vascular endothelial growth factor; FGF: Fibroblast endothelial growth factor; VCAM-1: Vascular cell adhesion molecule-1; ICAM-1: Intercellular adhesion molecule-1; PECAM-1: Platelet endothelial cell adhesion molecule-1; LFA: Lymphocyte function-associated antigen; IL-1ra: IL-1 receptor antagonist; GITR: Glucocorticoid-induced TNFR-related protein; CTLA4: Cytotoxic T-Lymphocyte Antigen 4; EMPs: Endothelial microparticles; COUP-TFII: Chicken ovalbumin upstream promoter-transcription factor II; EndMT: Endothelial-mesenchymal transition.

## Competing interests

The authors declare that they have no competing interests.

## Authors’ contributions

JM carried out the primary literature search and drafted the manuscript. AV and JS provided material input and helped revise the manuscript. HW and XFY conceived the study and provided field expertise. All authors read and approved the final manuscript.
